# Characterization of Antimicrobial Poly (Lactic Acid)/Nano-Composite Films with Silver and Zinc Oxide Nanoparticles

**DOI:** 10.3390/ma10060659

**Published:** 2017-06-16

**Authors:** Zhuangzhuang Chu, Tianrui Zhao, Lin Li, Jian Fan, Yuyue Qin

**Affiliations:** 1 Institute of Yunnan Food Safety, Kunming University of Science and Technology, Kunming 650550, China; 18487262163@163.com (Z.C.); food363@163.com (T.Z.); 2 College of Light Industry and Food Science, South China University of Technology, Guangzhou 510640, China; felinli@scut.edu.cn

**Keywords:** poly (lactic acid), nanoparticle, thermal properties, mechanical property, antimicrobial property

## Abstract

Antimicrobial active films based on poly (lactic acid) (PLA) were prepared with nano-silver (nano-Ag) and nano-zinc oxide (nano-ZnO) using a solvent volatilizing method. The films were characterized for mechanical, structural, thermal, physical and antimicrobial properties. Scanning electron microscopy (SEM) images characterized the fracture morphology of the films with different contents of nano-Ag and nano-ZnO. The addition of nanoparticles into the pure PLA film decreased the tensile strength and elasticity modulus and increased the elongation of breaks—in other words, the flexibility and extensibility of these composites improved. According to the results of differential scanning calorimetry (DSC), the glass transition temperature of the PLA nano-composite films decreased, and the crystallinity of these films increased; a similar result was apparent from X-ray diffraction (XRD) analysis. The water vapor permeability (WVP) and opacity of the PLA nano-composite films augmented compared with pure PLA film. Incorporation of nanoparticles to the PLA films significantly improved the antimicrobial activity to inhibit the growth of *Escherichia coli*. The results indicated that PLA films with nanoparticles could be considered a potential environmental-friendly packaging material.

## 1. Introduction

In last few decades, plastic bags made of polyethylene (PE), low-density polyethylene (LDPE), high-density polyethylene (HDPE), and polypropylene (PP) have become common in daily life [1]. These polymers are non-biodegradable and use petroleum as their raw material, and the environment can be contaminated with these bags [2]. Therefore, biodegradable and nontoxic packing materials have a huge expanding area on the market. Poly (lactic acid) (PLA) is a biodegradable polymer which can be produced from the bacterial fermentation of renewable resources, like sugar beet or corn starch [3,4]. PLA has been approved by the United States Food and Drug Administration (FDA). In addition, PLA has other advantages, like moderate mechanical properties, transparency, commercial availability and low price [5,6,7,8]. As a consequence, these advantages give PLA the best potential for packaging and medical applications [6,8]. However, some properties of PLA, such as flexural properties, gas and water vapor permeability and thermal stability, are not sufficient for certain applications [9]. In recent years, many studies have been reported on improving some properties of PLA by blending it with diversified additives, like plasticizer, other polymers and nanoparticles [9,10,11,12]. In our previous study, we tried to prepare PLA composite films with nano-silver (nano-Ag) and nano-zinc oxide (nano-ZnO).

Ag has been known to have antibacterial effects since ancient times [13], and recently, Ag has been used to control bacterial growth in many areas of applications [14,15,16]. Ag ions, even a low concentration, have a very broad range of activity against microorganisms like bacteria, yeast, fungi, and viruses [17,18,19,20], and low toxicity to human cells [21]. Nevertheless, the synthesis of nano-Ag with reduction in its size, affects its chemical and antibacterial properties considerably [22]. To solve this problem, nano-Ag can be blended with polymers like PLA to form nano-composites [9], or blended with a layer of metal-oxide, such as zinc oxide [23] and titanium dioxide [24], to form core-shell morphologies that offer large surface area to volume ratio.

ZnO nanoparticles have the advantage of low cost, easy availability, antibacterial effects, and intensive ultraviolet absorption [25]. Moreover, nano-ZnO has been approved by the FDA as a safe material [23]. Hence, as a functional inorganic important material, nano-ZnO is increasingly being developed for use in research and health-related applications [26,27].

In this work, antimicrobial and degradable PLA nano-composite films with different contents of nanoparticles were prepared using a solvent volatilizing method. The objective of this study was to evaluate the effects of these blend films on thermal, morphological, mechanical, gas barrier, and antimicrobial properties. Meanwhile, the structure-property relationship for PLA nano-composite films was also analyzed.

## 2. Results

### 2.1. X-ray Diffraction

**Figure 1:**
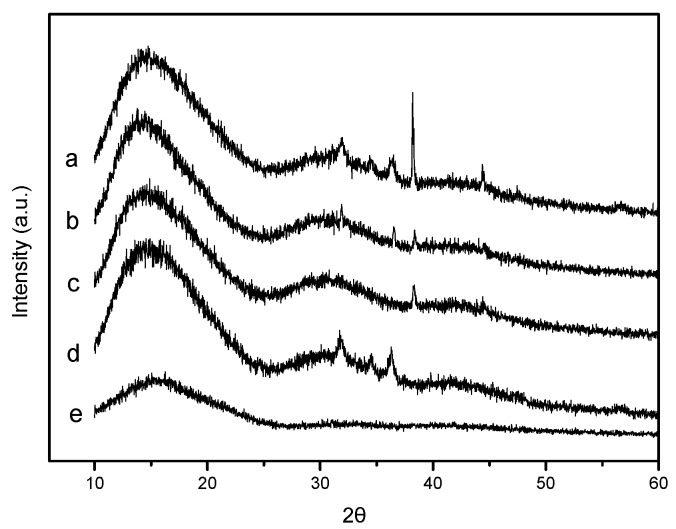
The X-ray diffraction (XRD) patterns of: (**a**) Poly (lactic acid) (PLA)/Ag/ZnO-3; (**b**) PLA/Ag/ZnO-1; (**c**) PLA/Ag; (**d**) PLA/ZnO; and (**e**) PLA.

The structures of pure PLA and PLA nano-composite films were analyzed by X-ray diffraction (XRD) to study the effect of nanoparticle content on the crystallinity of the PLA matrix. The XRD patterns of PLA and PLA nano-composite films are shown in Figure 1a–e. All the films showed intensity with a broad maximum appearing at approximately 2θ = 16°, which confirmed that the PLA had no polymorphic crystalline transition [28]. For PLA/Ag/ZnO blend films (Figure 1a–c), it was observed that with the increase in the nano-ZnO content (0, 1, and 3 wt %) the intensity of diffraction peaks at approximately 2θ = 31.8°, 2θ = 34.4° and 2θ = 36.3° increased, and these diffraction angles were consistent with the standard properties for hexagonal ZnO crystals (Joint Committee for Powder Diffraction Studies (JCPDS) No. 36-1451) [29]. Furthermore, when compared with a PLA/ZnO film (Figure 1d), the above diffraction peaks reduced or disappeared. This might have been due to the decline of nano-ZnO content [12]. In addition, the diffraction peaks (Figure 1a–c) evidenced at 2θ = 38.3° and 2θ = 44.4° fit the standard properties for FCC silver (JCPDS, No. 04-0783) [30], and the strength of these peaks were inconsistent. This could have been due to the addition of nano-ZnO with different contents [23].

### 2.2. Fourier Transform Infrared Spectra

Fourier transform infrared (FTIR) spectra were measured for each film to study their chemical structures. The principle pure PLA absorption bands in the infrared range were summarized in Table 1. The infra-red (IR) spectra at 2996.5 and 2977.9 cm^−1^ were assigned to the asymmetric and symmetric CH stretching region of the –CH3 mode, respectively. The C=O stretching of the ester group was attributed as a broad and strong absorption band at 1747.4 cm^−1^. The region between 1500 and 1360 cm^−1^ was characterized by the 1452.3 cm^−1^ CH_3_ band. The CH deformation and asymmetric bands appeared at 1382.9 cm^−1^ and 1359.7 cm^−1^, respectively. Between 1300 cm^−1^ and 1000 cm^−1^, it was possible to observe the C=O stretching mode of the ester group at 1267.1 cm^−1^ and the asymmetric C–O–C stretching mode at 1180.4, 1128.2, and 1080.1 cm^−1^. In the region of 1000 cm^−1^ and 800 cm^−1^, the band at 956.2 cm^−1^ was attributed to the characteristic vibration of the helical backbone with CH_3_ rocking mode. Two bands related to the crystalline and amorphous phases of PLA were found at 867.1 and 753.5 cm^−1^. The peak at 867.1 cm^−1^ could be assigned to the amorphous phase and the peak at 753.5 cm^−1^ to the crystalline phase [8,31,32].

**Figure 2:**
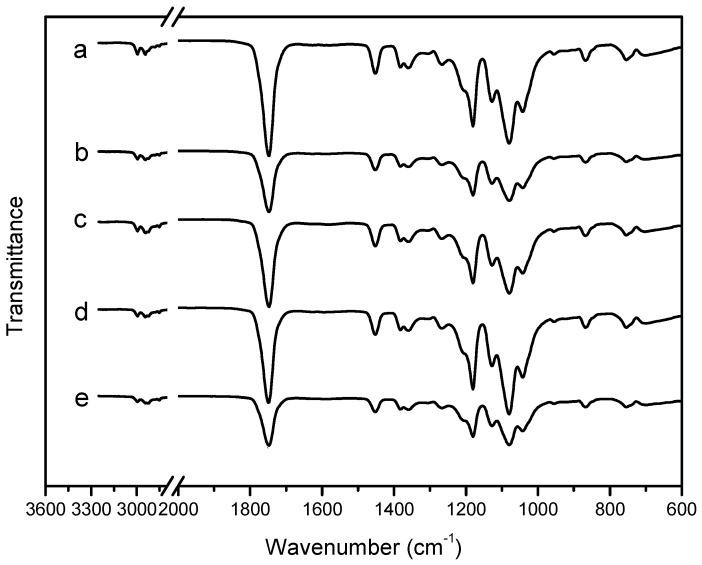
The Fourier transform infrared (FTIR) spectra of: (**a**) PLA/Ag/ZnO-3; (**b**) PLA/Ag/ZnO-1; (**c**) PLA/Ag; (**d**) PLA/ZnO; and (**e**) PLA.

FTIR spectra of the pure PLA and PLA nano-composite films are shown in Figure 2a–e. As shown in Figure 2, the curves of PLA nano-composite films (Figure 2a–d) were similar to the pure PLA film. However, with the addition of nanoparticles, the band, at 1747.4, 1452.3, 1180.4, and 1080.1 cm^−1^, increased in intensity. This may have occurred because the nucleation rate and nucleation density of PLA was increased by addition of nanoparticles.

### 2.3. Scanning Electron Microscopy

**Figure 3:**
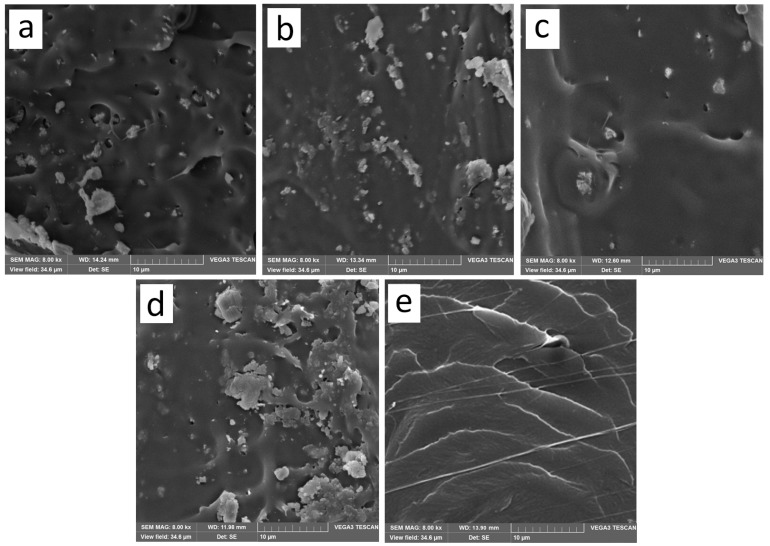
Scanning electron microscopy (SEM) micrographs of the fracture morphology of: (**a**) PLA/Ag/ZnO-3; (**b**) PLA/Ag/ZnO-1; (**c**) PLA/Ag; (**d**) PLA/ZnO; and (**e**) PLA.

Scanning electron microscopy (SEM) images of cross-sectional microstructures of the blended films were shown in Figure 3a–e. From Figure 3, we could see that the cross-section of the pure PLA film was a stratified structure, and by adding the nanoparticles, the microstructures were changed. As shown in Figure 3a–c, the stratified structure gradually disappeared and some small voids appeared on the fractured surface of PLA/Ag/ZnO blend films, and more voids formed with the increase of the nano-ZnO content. Furthermore, the higher the content of nano-ZnO, the more white particles aggregated. These aggregates indicated that the incomplete dispersion/blend of the nanoparticles at different content in the bulk of all films.

### 2.4. Mechanical Properties

Mechanical parameters such as elasticity modulus (EM), tensile strength (TS), and elongation of break (E) are important factors in evaluating polymeric films. The mechanical properties of PLA films containing nanoparticles are shown in Table 2. For the pure PLA film, EM, TS and E were measured to be 3118.8 MPa, 47.8 MPa, and 5.35%, respectively. Furthermore, from Table 2, it could be seen that addition of nanoparticles reduced the EM and TS values of the films and increased the E value of the films compared with pure PLA film. The purpose of blending nanoparticles with PLA was to improve the plastic elongation and lower the brittleness.

The EM and TS values of PLA nano-composite films slightly changed when compared to pure PLA film. From Table 2, the EM values of PLA/Ag/ZnO blend films decreased by approximately 9.84%, 29.32%, and 18.94% upon incorporation of 0, 1, and 3 wt % of nano-ZnO content, respectively. PLA/ZnO film decreased by about 16.29%. For the TS values, the PLA nano-composite films fell to 28.16 ± 2.69 MPa from 44.30 ± 3.62 MPa. The PLA/Ag/ZnO-3, PLA/Ag/ZnO-1 and PLA/ZnO films caused a significant (*p* < 0.05) reduction in the EM and TS values. This result may have been due to the addition of antimicrobial agents, which decreased the interactions between PLA chains and enhanced the mobility of PLA chains [33]. However, a slight reduction in EM and TS values was observed to PLA/Ag/ZnO-3 when compared to that which was incorporated with 1 wt % nano-ZnO. This may have been due to the molecular weight diminished and low molecular products formed.

The values of E of PLA/Ag/ZnO blend films was improved by 24.86%, 128.41%, and 57.01% upon incorporation of 0, 1, and 3 wt % of nano-ZnO content, respectively. PLA/ZnO film increased about 44.67%. This increase in the E value may have occurred because some plasticizing effect caused by the addition of nanoparticles to the polymer matrix resulting in the increase in ductile properties, which would also result in changes in the crystallinity of polymer [34]. It could be seen that the E values did not increase with the increase of content of nano-ZnO. This might be because that nano-ZnO concentration exceeded the maximum concentration allowed for forming homogeneous composites [35]. Although the value of E increased compare with pure PLA film, it still low. To improve the mechanical properties in the further applications, the addition of plasticizer into PLA nano-composite films can be supplementary considered.

### 2.5. Differential Scanning Calorimetry

**Figure 4:**
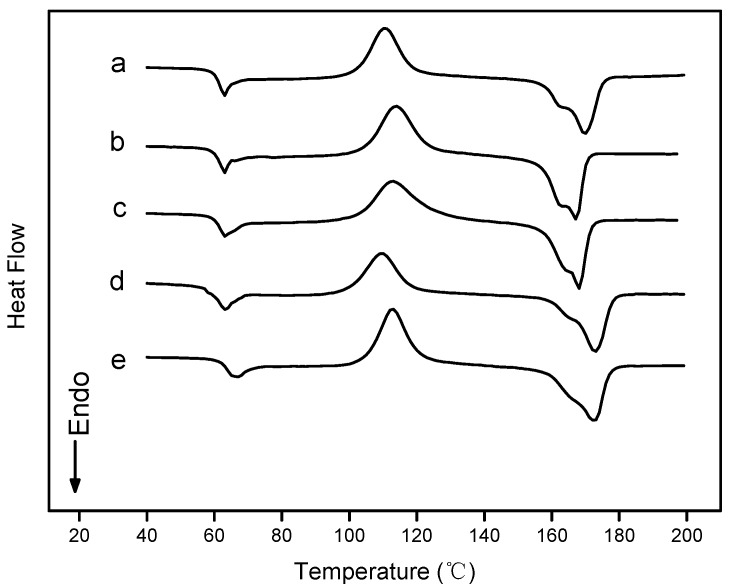
Differential scanning calorimetry (DSC) curves of: (**a**) PLA/Ag/ZnO-3; (**b**) PLA/Ag/ZnO-1; (**c**) PLA/Ag; (**d**) PLA/ZnO; and (**e**) PLA.

Differential scanning calorimetry (DSC) was used to study the glass transition, melting and crystallization of pure PLA and PLA nano-composite films. DSC heating thermograms of pure PLA and PLA nano-composite films were shown in Figure 4. DSC thermal properties such as the glass transition temperature (*T_g_*), melting temperature (*T_m_*) and crystallinity (*X_c_*) are summarized in Table 3. The *T_g_* values of PLA nano-composite films were very small, and approximately 3 °C lower than the pure PLA film. In addition, small endothermic peaks were displayed within this temperature range for the pure PLA and PLA nano-composite films, this might have been due to the typical effect of physical aging [36]. The *T_c_* values slightly decreased with the addition of nanoparticles, whereas they decreased by 5.8 °C in PLA/ZnO. This may have been due to heterogeneous nucleation, and nanoparticles in PLA acted as the heterogeneous nucleus and promoted the cold crystallization of PLA chains at lower temperatures [37]. The obvious peak at one of the highest temperature was regarded as the melting temperature (*T_m_*) of all the films. The *T_m_* values of PLA nano-composite films were lower than pure PLA films. As was shown in Figure 4, there were double melting peaks near the *T_m_*. This double peak could be explained as the result of annealing occurring during the DSC scans whereby crystals had time to recrystallize a few degrees above the melting point and then remelt [37]. When added the nanoparticles, the double melting peaks became clearer. The lower *X_c_* (7.1–18.8) of pure PLA and PLA nano-composite films was shown Table 3. However, amorphous regions of PLA, which were affected by degradation, decomposed faster with lower *X_c_* [38]. The DSC heating process was essentially an annealing process. The *X_c_* could be improved by annealing according to the requirements of the biodegradation rate and the mechanical properties for the applications. All of these results suggested that the nanoparticles could promote crystallization of PLA, including cold crystallization and re-crystallization.

### 2.6. Water Vapor Permeability

**Figure 5:**
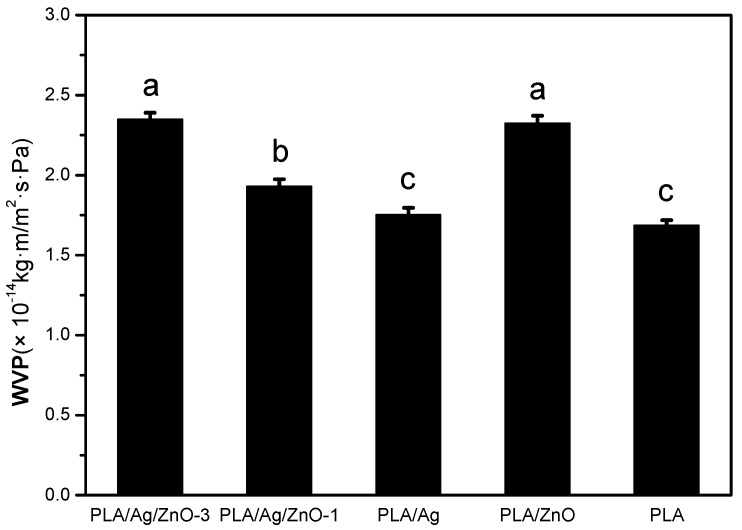
Water vapor permeability (WVP) analysis of pure PLA and PLA nano-composite films. Values followed by different superscript letters (a–c) in the same column were significantly different (*p* < 0.05), where a is the highest value.

Water was a noticeable factor in deterioration reactions and microbial growth of food, so minimize even avoid the moisture transfer between food and the surrounding atmosphere became important. In other words, Water vapor permeability (WVP) was one of the most important properties in food packaging applications. The WVP values for pure PLA and PLA nano-composite films were shown in Figure 5. The WVP of pure PLA film was 1.69 × 10^−14^ kg m/m^2^ s Pa. The WVP of PLA/Ag/ZnO blend films increased significantly (*p* < 0.05) with the increase of nano-ZnO content from 1 to 3 wt %. This behavior may have been due to the hydrophilicity of nano-ZnO improving the hydrophilic interaction of films. On the other hand, the morphological structures of PLA blend films changed. This could be verified by SEM analysis showing that many voids in PLA/Ag/ZnO blend films and allowed more water vapor transfer. Although there was no significance difference in WVP between pure PLA film and PLA/Ag film (*p* > 0.05), the WVP value of PLA/Ag film was equal to the pure PLA film in the limit of measurement error. The addition of nanoparticles enhanced the flexibility and decreased the barrier properties of the PLA films. Similar results had been studied by other researchers. The WVP values of PLA blend films were increased by the addition of other agents like nanosilica [39] and nano-Ag [9]. In previous studies, the high WVP of film led to a slight decline in relative humidity within the packaging, which would be beneficial for postharvest vegetable and fruit quality. Furthermore, there was no condensate water when using materials with high WVP. This would help to reduce microbial growth and maintain an acceptable appearance of postharvest mushrooms [40]. Therefore, the PLA nano-composite films could be potentially be used for food packaging applications.

### 2.7. Opacity

**Figure 6:**
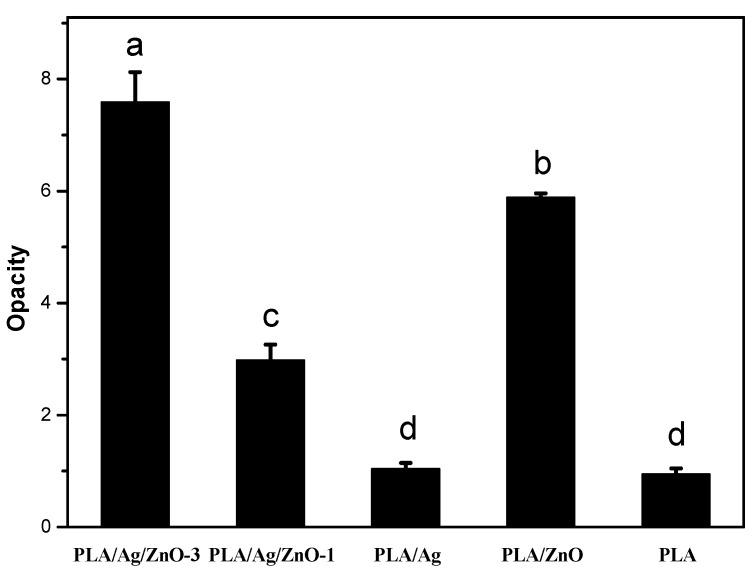
Opacity analysis of pure PLA and PLA nano-composite films. Values followed by different superscript letters (a–d) in the same column were significantly different (*p* < 0.05), where a is the highest value.

**Figure 7:**
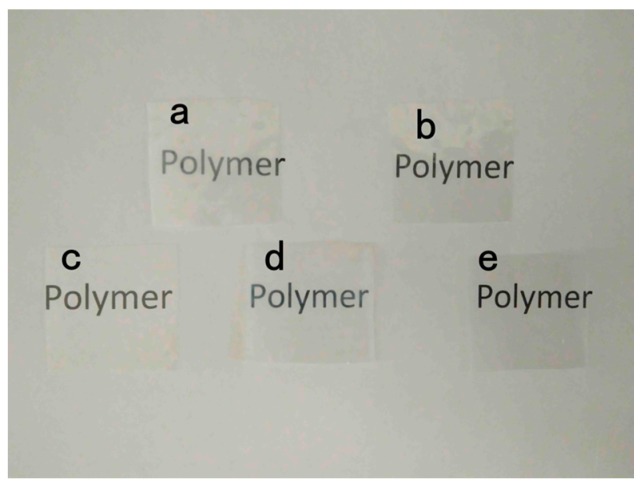
The visual appearance of: (**a**) PLA/Ag/ZnO-3; (**b**) PLA/Ag/ZnO-1; (**c**) PLA/Ag; (**d**) PLA/ZnO; and (**e**) PLA.

To a large extent, the appearance of goods influences the consumers to purchase them, so the transparency of packaging is important. The opacity of the pure PLA and PLA nano-composite films is shown in Figure 6. The transparency of PLA nano-composite films was lower than the pure PLA film. The opacity values of the PLA/Ag/ZnO-3, PLA/Ag/ZnO-1 and PLA/ZnO blend films were significantly (*p* < 0.05) higher than pure PLA and PLA/Ag films. This may have been because of the color and the content of nanoparticles. The decrease in film transparency with PLA-base films by adding the antimicrobial agents had also been reported [34]. The visual appearance of pure PLA and PLA nano-composite films is shown in Figure 7. Statistically significant differences were found between samples with nanoparticles, but differences in visual appearance were not notable to the human eye. This result suggested that, for consumers, there was no visual influence and people could see items sufficiently through the films when these films were applied to the packaging applications.

### 2.8. Antimicrobial Activity

**Figure 8:**
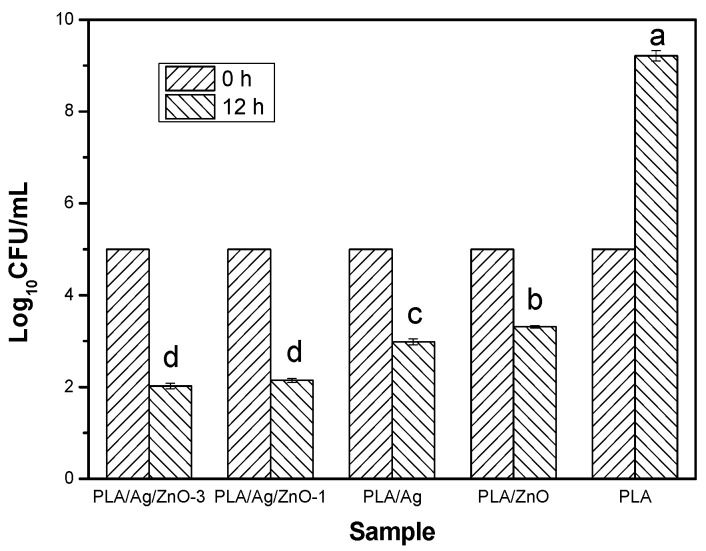
Antimicrobial activity of pure PLA and PLA nano-composite films. Values followed by different superscript letters (a–e) in the same column were significantly different (*p* < 0.05), where a is the highest value.

To evaluate the antimicrobial properties of nanoparticles nano-ZnO and nano-Ag, the PLA nano-composite films was studied for inhibition of *E. coli* growth, which was chosen in this study as a representative of common harmful microorganisms occurring in food products. The results of the antimicrobial test are shown in Figure 8. After 12 h of the experiment, the control film, the pure PLA film, had no antimicrobial activity to inhibit the growth of *E. coli*. The Log_10_CFU/mL value for pure PLA film was up to approximately 9.21 from 5 at 0 h of the experiment. On the contrary, the Log_10_CFU/mL values of the PLA nano-composite films with antimicrobial nanoparticles shown to be significantly lower than the pure PLA (*p* < 0.05), and the values 2.02, 2.14, 2.98 and 3.31 Log_10_CFU/mL represented the PLA/Ag/ZnO-3, PLA/Ag/ZnO-1, PLA/Ag and PLA/ZnO films, respectively at the end of 12 h of the experiment. These inhibitions were presumably due to the addition of nanoparticles. These nanoparticles released the surface of films through the micro-voids, formed in the PLA nano-composite films by the nanoparticles, and restrained the growth of *E. coli* under the present experimental condition [41]. On the one hand, the Log_10_CFU/mL value of the PLA/Ag film was significantly (*p* < 0.05) less than the PLA/ZnO film. This may have been due to the different antimicrobial mechanisms of nano-ZnO and nano-Ag, and the low-dose of nano-Ag could be against the bacteria [17]. On the other hand, the PLA/Ag/ZnO films with a nano-ZnO content from 1 to 3 wt % were significantly lower than the other nano-composite films (*p* < 0.05). This may have been through the synergy between nano-ZnO and nano-Ag improving the antimicrobial activity of nano-composite films when these nanoparticles were added to the PLA films. In fact, the ability for the nano-composite films to inhibit other microorganisms on various food products at various storage temperatures should be tested in the future.

## 3. Materials and Methods

### 3.1. Materials and Chemicals

Poly (lactic acid) (PLA, Mw = 280 kDa, Mw/Mn = 1.98) was purchased from Natureworks LLC (Blair, NE, USA). Nano-Ag was purchased from Jinda Nano Tech Co., Ltd (Xiamen, Fujian, China). Nano-ZnO was purchased from Qingdao nakasen Zinc & Technology Co., Ltd (Qingdao, Shandong, China). All other reagents and chemicals used in the study were analytical grade.

### 3.2. Preparation of Films

Pure PLA and PLA nano-composite films were prepared by the solvent volatilizing method. Before preparation, the PLA was dried in a vacuum oven at 80 °C for 12 h to eliminate the influence of water. A total of 2 g PLA was dissolved in 50 mL dichloromethane and stirred for 12 h. Then, different amounts of nano-Ag or nano-ZnO was added to the PLA dichloromethane solution and stirred by a magnetic stirrer until all of the nanoparticles were completely mixed. Subsequently, the solutions were cast onto polytetrafluoroethylene (PTFE) plates and dried in a vacuum oven at room temperature. The PLA film with 3 wt % nano-ZnO was named as PLA/ZnO, and with 0.5 wt % nano-Ag was named as PLA/Ag. Nano-ZnO was incorporated into PLA/Ag as 1 and 3 wt % loading named as PLA/Ag/ZnO-1 and PLA/Ag/ZnO-3, respectively. The pure PLA film was used as control.

### 3.3. X-ray Diffraction

The structure of nano-composites was evaluated by XRD, via an X-ray diffractometer (D8 Advance, Brucker, Karlsruhe, Germany) with Cu Kα radiation, at a voltage of 40 kV and an electricity of 40 mA. The samples were scanned in the diffraction angle 2θ, with a scan speed of 2°/min at room temperature.

### 3.4. Fourier Transform Infrared Spectra

FTIR measurements were accomplished using an FTIR spectrometer (Nicolet-5700, Ametek, Inc., Shanghai, China) in the range of 400–4000 cm^−1^ at a resolution of 4 cm^−1^.

### 3.5. Scanning Electron Microscopy

The cross-section morphology of the blend films was performed with a scanning electron microscope (NOVA NanoSEM 450, FEI, Hillsboro, OR, USA). Before the SEM examination, the films were submerged in liquid nitrogen and fractured. The fracture surfaces of the films were coated with a thin conductive gold layer in 20 nm thick.

### 3.6. Mechanical Properties

TS, E, and EM were measured with a Universal tensile machine (CMT 4104, MTS Systems Co., Ltd., Shenzhen, China) following the American Society for Testing and Materials (ASTM) Standard Method D 882-88. The initial grip separation was set at 100 mm and the crosshead speed was set at 50 mm/min. The mechanical tests were replicated five times for each type of film.

### 3.7. Differential Scanning Calorimetry

DSC measurements were performed with a TA Instruments (DSC 214, Netzsch, Selb, Germany) under nitrogen atmosphere. Firstly, the sample was sealed in an aluminum crucible and heated from 20 °C to 200 °C at a heating rate of 10 °C/min, and held for 5 min to eliminate the previous thermal history. Then the sample was cooled at 10 °C/min to 20 °C. Subsequently, the sample was heated again from 20 °C to 200 °C at a heating rate of 10 °C/min. The second heating scan was used to evaluate melting temperature (*T_m_*) and crystallinity (*X_c_*) of the samples. The percentage of crystallinity (*X_c_*) for sample was calculated according to the following formula [42].

(1) $$X_{c}\left(\%\right)=\frac{\Delta H_{m}-\Delta H_{c}}{\Delta H_{m}^{o}\times w}\times 100,$$

where Δ*H_m_* is the melting enthalpy (J/g) of PLA in the sample, Δ*H_c_* is the cold crystallization enthalpy (J/g) of PLA in the sample, Δ$H_{m}^{o}$ is the heat of fusion for completely crystalline PLA (93.7 J/g) [43], and w is the weight fraction of PLA in the samples.

### 3.8. Water Vapor Permeability

Water Vapor Permeability (WVP) of films was determined gravimetrically according to the ASTM E96-95 standard method [44]. Previously, the test films were placed in the environment at 25 °C and 50% relative humidity (RH) for 48 h. Then, weighing bottles were filled with allochroic silicagel and the test films were sealed on the bottles by using paraffin and rubber. The covered bottles were put in a temperature and RH controlled chamber to equate the conditions of test. The weight loss of each bottle was measured as a function of time for 12 h. The WVP of the film was calculated with the following formula [45].

(2) $$\mathrm{WVP}=\frac{WVTR\times L}{\Delta P},$$

where *WVTR* is the water vapor transmission rate (g/m^2^ s) through the film, *L* is the average film thickness (m), and Δ*P* is the water vapor pressure difference (Pa) between the two sides of the film. This entire test was repeated in triplicate for each type of film.

### 3.9. Opacity

Opacity was determined by measuring the absorbance at 600 nm using a UV-Vis spectrophotometer (T90, Beijing Purkinje General Instrument Co., Ltd., Beijing, China) [46]. Each sample was cut into a rectangle section (0.7 cm × 1.5 cm) and directly placed in a spectrophotometer test cell. An empty test cell was used as the reference. The film opacity was calculated as following formula.

(3) $$\mathrm{Opacity}=\frac{Abs_{600}}{d},$$

where *Abs*_600_ is the value of absorbance at 600 nm and d is the film thickness (mm). This test was triplicated for each type of film.

### 3.10. Antimicrobial Activity

The liquid culture test was used to evaluate the antimicrobial activity of PLA nano-composite films. In other words, this test measured the ability of nanoparticles to restrain the growth of the food pathogenic bacteria *Escherichia coli*. *E. coli* was obtained Laboratory of Microbiology, Faculty of Life Science and Technology, Kunming University of Science and Technology, Yunnan, China. *E. coli* was activated by inoculation into Mueller-Hinton broth. The bacteria were incubated at 37 °C for 18–24 h. A glass test tube containing a testing sample (0.18–0.20 g) was filled with 10 mL of broth, and inoculated with 0.1 mL of an overnight culture of *E. coli*. The bacterial cultures concentration was adjusted to 10^5^ CFU/mL. And these test tubes were shaken at 150 rpm at 22 °C. After 0 and 12 h of the experiment, aliquots containing 1 mL of sample were serially diluted with sterile phosphate buffer (pH = 7.4) and then plated onto petri dishes. All petri dishes were incubated at 37 °C for 24 h and colony-forming units (CFU) were counted [41]. The pure PLA film was used as control.

### 3.11. Statistical Analysis

The date were represented as the mean ±standard deviations and analysis of variance (ANOVA) using SPSS (SPSS Inc., version 19.0, Chicago, IL, USA). Duncan’s multiple range test method was applied to compare means for each test, with the statistical significance was defined at a level of *p* < 0.05.

## 4. Conclusions

The work was focused on the key-characteristics of pure PLA and PLA nano-composite films. PLA nano-composite films were prepared with nano-ZnO and nano-Ag by solvent volatilizing method. According to DSC and consistent with XRD, which were demonstrated that the pure PLA and PLA nano-composite films were mostly amorphous. FTIR analysis found that there were strong interactions between PLA and nanoparticles. SEM analysis showed that the incompatibility of PLA and the addition of nanoparticles in blends influenceed the morphology. The addition of nanoparticles into the pure PLA film decreased the TS and EM, and increased the elongation of breaks, that is, the flexibility and extensibility of the films improved. WVP and opacity properties of films were higher by the addition of nanoparticles. In this study, the PLA nano-composite films showed good antimicrobial activity against *E. coli*, especial the PLA/Ag/ZnO-3 and PLA/Ag/ZnO-1. In brief, the PLA nano-composite films might have good potential for the application of antimicrobial packaging. Further research to improve the plasticity of the composite films by adding plasticizer or essential oils with plasticization will have further applications for the food packaging field.

## Figures and Tables

**Table 1:** Peak band assignments of pure PLA and PLA nano-composite films spectrums.

Assignment	Peak Position (cm^−1^)
–CH– stretch	2996.5 (asym), 2977.9 (sym)
–C=O carbonyl stretch	1747.4
–CH_3_ bend	1452.3
–CH– deformation symmetric and asymmetric bend	1382.9, 1359.7
–C=O bend	1267.1
–C–O– stretch	1180.4, 1128.2, 1080.1
–OH bend	1041.5
–CH_3_ rocking modes	956.2
–C–C– stretch	867.1

**Table 2:** The mechanical properties of pure PLA and PLA nano-composite films.

Sample	Elasticity Modulus (EM)	Tensile Strength (TS)	Elongation of Break (%)
PLA/Ag/ZnO-3	2528.20 ± 223.54 ^bc^	36.15 ± 5.55 ^c^	8.40 ± 0.11 ^b^
PLA/Ag/ZnO-1	2204.31 ± 297.81 ^c^	28.16 ± 2.69 ^d^	12.22 ± 0.12 ^a^
PLA/Ag	2811.76 ± 167.26 ^ab^	44.30 ± 3.62 ^ab^	6.68 ± 0.10 ^d^
PLA/ZnO	2610.64 ± 297.51 ^b^	38.96 ± 8.19 ^bc^	7.74 ± 0.17 ^c^
PLA	3118.79 ± 333.39 ^a^	47.78 ± 5.18 ^a^	5.35 ± 0.06 ^e^

Values followed by different superscript letters (a–e) in the same column were significantly different (*p* < 0.05), where a is the highest value.

**Table 3:** Thermal characteristics of pure PLA and PLA nano-composite films.

Sample	Glass Transition Temperature *T_g_* (°C)	Crystallization Temperature *T_c_* (°C)	Melting Temperature *T_m_* (°C)	Crystallinity *X_c_* (%)
PLA/Ag/ZnO-3	59.3	110.5	169.7	16.2
PLA/Ag/ZnO-1	60.9	112.6	167.8	18.8
PLA/Ag	60.4	111.7	169.0	13.6
PLA/ZnO	60.2	107.5	170.7	15.3
PLA	63.6	113.3	173.2	7.1
